# Utilization of gold nanoparticles for the detection of squamous cell carcinoma of the tongue based on laser-induced fluorescence and diffuse reflectance characteristics: an in vitro study

**DOI:** 10.1007/s10103-022-03634-9

**Published:** 2022-08-24

**Authors:** Maha Nour, Omnia Hamdy, Amna H. Faid, Elsayed Abdallah Eltayeb, Ahmed Abbas Zaky

**Affiliations:** 1 Department of Medical Applications of Laser, National Institute of Laser Enhanced Sciences (NILES), Cairo University, Giza, 12613 Egypt; 2 Department of Engineering Applications of Lasers, National Institute of Laser Enhanced Sciences (NILES), Cairo University, Giza, 12613 Egypt; 3 Department of Laser Science and Interaction, National Institute of Laser Enhanced Sciences (NILES), Cairo University, Giza, 12613 Egypt

**Keywords:** Oral cancer; Squamous cell carcinoma, Laser-induced fluorescence; Reflectance, Gold nanoparticles

## Abstract

Squamous cell carcinoma is a very common type of oral cancer that affects the health of people with an unacceptably high mortality rate attributed to the difficulties in detecting the disease at an early stage. Therefore, effective techniques for early diagnosis and effective therapy of oral cancer are necessary. In the present study, we exploit the ability of gold nanoparticles (AuNPs) to undergo coupled surface plasmon resonance when closely spaced to improve diagnosing squamous cell carcinoma of the tongue. The prepared AuNPs are characterized by UV–VIS spectroscopy, dynamic light scattering, Fourier transform infrared spectroscopy, and transmission electron microscopy. The size of the prepared AuNPs is 12 ± 2 nm with narrow size distributions and exhibited high stability with a zeta potential of − 16.5 mV. The light fluorescence of the normal and cancer cells is recorded before and after NP addition using a spectrometer upon excitation by 405-nm laser irradiation. Furthermore, the light reflectance of the examined samples is measured at different laser wavelengths (red to NIR region). The obtained results show that the cancer cells mixed with AuNPs produce a higher fluorescence peak at 489.2 nm than the cancer cells without AuNPs. Moreover, the optical diffuse reflectance analyses reveal that the addition of AuNPs enhances cancer detection especially at the 635-nm irradiation with sensitivity (94%), specificity (87%), and overall accuracy (91%).

## Introduction

Squamous cell carcinoma (SCC) is one of the most common oral cavity cancers in the world [1]. However, the majority of these types of malignant tumors occur in the tongue especially in the lateral part [2]. They are characterized by early lymph node metastasis, rapid local invasion, a pain-burning sensation of the tongue, and poor prognosis [3]. Smoking and drinking are considered risk factors that can increase the chance of having such type of cancer [4]. Most of the early stages of oral cancer patients are not symptomatic; therefore, they do not seek medical examination until the appearance of clear symptoms such as bleeding, plan, or amass in the mouth or neck which occurs if the lymphatic spread has already present [5]. Consequently, it is usually diagnosed in advanced stages (III and IV) with less than 40% survival rate [6]. Therefore, early detection of oral cancer is intensively needed.

Generally, oral carcinoma can be detected via many diagnosed tests and modalities, such as bone scan [7], computed tomography (CT) [8], magnetic resonance imaging (MRI) [9], and ultrasonography [10]. However, some cases are still suffering from poor prognosis and low detection and 5-year survival rates. Therefore, alternative and/or modified techniques are essential for the early detection of oral cancers. Nanotechnology is also a powerful tool for cancer detection, and monitoring. It has been applied in the detection and diagnosis of various cancers, such as cervical, lung, breast, gastric, naso-pharyngeal, colon, and oral cancer [11, 12]. Nanotechnology has improved cancer diagnosis by enhancing both screening and imaging techniques through the use of nanomaterials and nanofabrication which enable new detection platforms with enhanced properties for early detection of cancers. Gold nanoparticles (AuNPs) have a wide use in biomedical applications due to its unique properties that make them suitable for diagnosis and treatment of cancer. AuNPs also have low toxicity and are non-immunogenic by nature. Additionally, their synthesis methods are simple and their shape and surface modifications can be readily controlled [13].

Many optical diagnostic techniques have been developed for oral cancer early screening such as autofluorescence spectroscopy. In a typical light-induced fluorescence procedure, molecules in the examined sample are excited by the absorption of specific wavelength laser photons. Then, the excited molecules emit fluorescent light at longer wavelengths [14]. The emission bands of the resultant fluorescence spectrum are utilized to identify different molecular components of the sample. Such emission occurred due to the presence of specific fluorophores. Collagen, tryptophan, elastin, keratin, hemoglobin, and NADH are the main fluorophores in the oral cavity [15]. Oral cancer causes alterations in the concentration of such fluorophores which leads to alteration of light beam properties within the tissue; these changes in the spectral property of the mucosa can be detected and consider as a diagnostic tool in malignant disorders and cancerous conditions. In the present work, gold nanoparticles (AuNPs) were prepared by using trisodium citrate as a reducing and capping agent and used to improve the detection of basal cell carcinoma of the tongue. Laser source at 405 nm was used to excite the studied cells (normal and carcinoma) and the obtained fluorescence emission was recorded. Additionally, different laser wavelengths at visible and NIR spectral range have been interacted with the normal and cancer cells before and after adding AuNPs. For each case, the sample diffuse reflectance was recorded using a compact spectrometer.

## Material and methods

### Preparation of AuNPs by citrate reduction

The AuNPs were synthesized successfully according to a standard wet chemical method [16, 17], using trisodium citrate as a reducing and capping agent; 20 ml of 1 mM HAuCl_4_ solution was heated to boiling, refluxed while being stirred. Two milliliters of a 38.8 mM trisodium citrate solution was added quickly. The solution color changed from yellow to deep red. After changing the color, the heater was turned off and the solution was left to stir until reaching room temperature.

### Oral cell samples

Samples of oral epithelial cells (OEC) were obtained from Nawah Scientific Inc., (Mokatam, Cairo, Egypt). Cells were maintained in DMEM supplemented with 100 mg/ml of streptomycin, 100 units/ml of penicillin, and 10% of heat-inactivated fetal bovine serum in humidified, 5% (v/v) CO_2_ atmosphere at 37 °C. Twenty oral squamous cell carcinoma (cell line from ATCC) samples were collected from tongue (*Homo sapiens*, human Caucasian, male). The samples were kept at − 80 °C until the day of analysis. After thawing at room temperature, samples were divided into 4 groups as follows: group 1 is the normal cells (5 samples), group 2 is the normal cells mixed with AuNPs (5 samples), group 3 is the oral squamous cell carcinoma of the tongue (10 samples), and group 4 is the oral squamous cell carcinoma of the tongue mixed with AuNPs (10 samples). The second and the fourth groups were mixed in a 1:1 volume ratio with colloidal AuNPs of 12 ± 2 nm diameter acquired from Nawah Center (spherical nanoparticle with optical density 0.8). The characteristics of the studied cell lines are summarized in Table 1.

**Table 1:** The main characteristics of the utilized cell lines

Cell type	Normal epithelial cells	Squamous cell carcinoma
Organism	*Homo sapiens*, human	*Homo sapiens*, human
Tissue	Gingiva epithelium	Tongue
Product format	Frozen 1.0 ml	Frozen 1.0 ml
Morphology	Epithelial-like	Epithelial-like
Culture properties	Adherent	Adherent
Biosafety level	2	2
Age	Unknown	54
Gender	Male	Male
Ethnicity	Unknown	Caucasian

### Cytotoxicity assay

Cell viability was assessed by SRB assay [18, 19]. Aliquots of 100 μl cell suspensions (5 × 10^3^ cells) were in 96-well plates and incubated in complete media for 24 h. Cells were treated with another aliquot of 100-μl media containing AuNPs at various concentrations from 0.01 to 100 μg/ml. After 72 h of AuNP exposure, cells were fixed by replacing media with 150 μl of 10% TCA and incubated at 4 °C for 1 h. The TCA solution was removed, and the cells were washed 5 times with distilled water. Aliquots of 70-μl SRB solution (0.4% w/v) were added and incubated in a dark place at room temperature for 10 min. Plates were washed 3 times with 1% acetic acid and allowed to air-dry overnight. Then, 150 μl of TRIS (10 mM) was added to dissolve protein-bound SRB stain; the absorbance was measured at 540 nm using a BMG LABTECH®- FLUOstar Omega microplate reader (Ortenberg, Germany).

### UV–visible spectroscopic analysis

Absorption spectra of the synthesized AuNPs were monitored using a double-beam spectrophotometer (PG instrument, T80 + , UK). Two hundred microliters from the prepared solutions was diluted to 2 ml with distilled water then transferred to 1 cm UV-quartz cell and the absorption spectra were recorded within the appropriate scan range (200 to 800 nm).

### TEM examination

The morphology of the prepared AuNPs was investigated using TEM (Tecnai G20 FEI, Netherlands, super twin, double tilt, applied voltage is 200 kV, magnification range is up to 10^6^ × , and gun type is LaB6). Drops from a very dilute solution were deposited on an amorphous carbon-coated copper grid and left to evaporate at room temperature forming a monolayer then detected by TEM. All the investigations were performed at the Nanotechnology & Advanced Material Central Lab (NAMCL), Agriculture Research Center “ARC”, Giza, Egypt.

### Dynamic light scattering (DLS) analysis

The particle size and size distribution in terms of the average volume diameters and polydispersity index of the prepared particles were analyzed via photon correlation spectroscopy using a particle size analyzer, dynamic light scattering (DLS) (Zetasizer Nano ZN, Malvern Panalytical Ltd, UK) at fixed angle of 173° at 25° C. Samples were analyzed in triplicate. The same equipment was used for the determination of zeta potential.

### Fourier transform infrared (FTIR) spectroscopy analysis

FTIR measurements were carried out using a FTIR spectrometer (4100 Jasco-Japan) in the range 500–4500 cm^−1^. The prepared AuNPs were freeze-dried by lyophilization. IR spectra of powdered samples were diluted with a potassium bromide (KBr) pellet and then measured.

### Laser-induced fluorescence and reflectance measurement

**Fig. 1:**
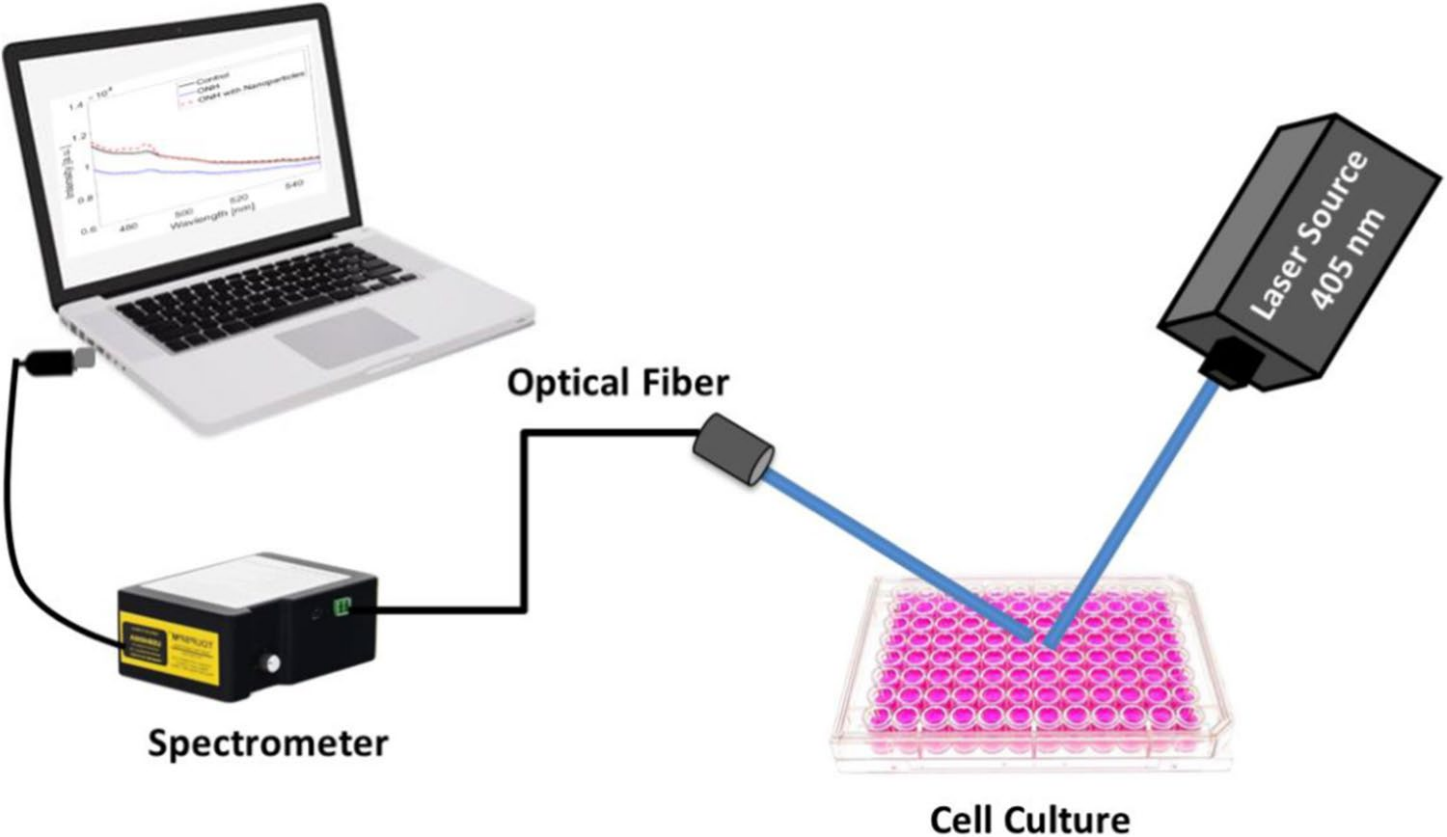
Schematic of the experimental setup used for collecting the fluorescence spectra

The optical setup for acquiring the fluorescence characteristics of normal and cancer cells is presented in Fig. 1. A continuous-wave diode pumped solid-state (DPSS) laser source (XL-R405SD, Xinland International Co., Ltd, China) at wavelength of 405 nm and output power of 100 mW has been used as the excitation source. The laser beam diameter is about 2 mm. Upon interacting with the tested oil samples, the resultant fluorescence emission has been delivered to a digital spectrometer (STDFSM, Touptek Photonics Co. Ltd, Zhejiang, China) via an optical fiber. The spectrometer is connected to a computer through a USB cable.

The measuring detector (Toshiba TCD1304AP Linear CCD array (Sony ILX511 2048 Linear CCD array)) has 200–1100 wavelength response range, 3648 pixels, and 8 × 200 µm pixel size. Data processing and analysis have been performed using the spectrometer software (Toup Spm) and Matlab R2018b. For measuring the optical diffuse reflectance of the samples, other laser wavelengths at the visible to NIR spectral region have been used (635, 785, 808, and 980 nm). The same optical setup presented in Fig. 1 is used but with the other laser sources.

## Results

### Size, zeta potential, and morphological analysis

**Fig. 2:**
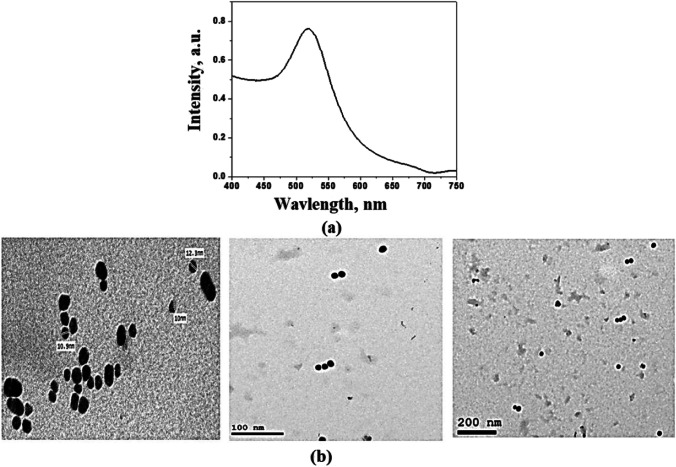
**a** UV–VIS absorption spectra and **b** TEM images of AuNPs

The prepared AuNPs show absorption in the visible range due to surface plasmon resonance (SPR) at 520 nm which indicates the stable state of the AuNPs clearly as presented in Fig. 2. The color of the AuNPs’ suspension was dark purple. The narrow band of the curve corresponds mostly to the monodisperse nature of AuNPs, without any aggregation and agglomeration [20, 21]. TEM observations were employed to clarify the morphology of formed AuNPs; the particles are spherical with approximate size (12 ± 2 nm) with uniform size distribution.

**Fig. 3:**
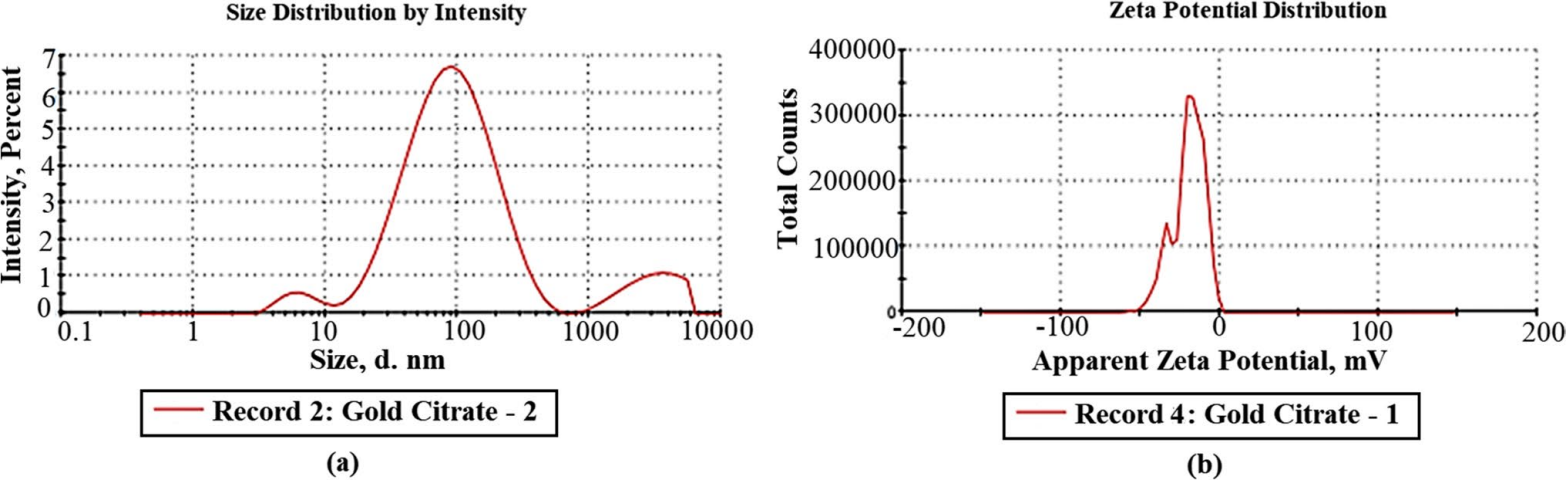
**a** Zeta size and **b** potential of AuNPs

Zeta potential and particle size are two important characteristics of nanoparticles (see Fig. 3). Size distribution and stabilization of particles were determined by a zetasizer. The mean particle size and zeta potential of AuNPs were measured to be 100 nm and − 16.5 mV, respectively.

### FTIR analysis

**Fig. 4:**
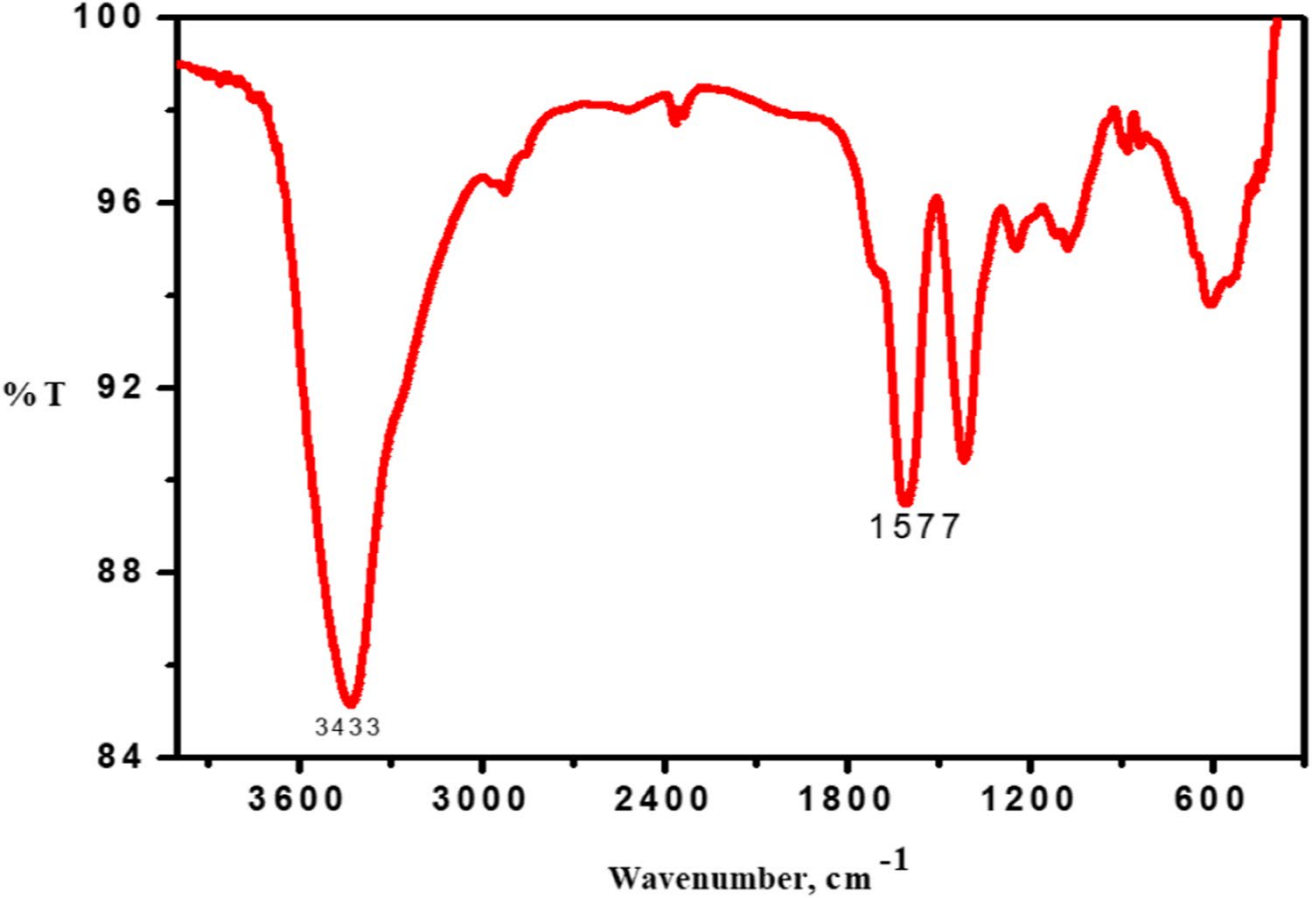
FTIR spectra of the utilized AuNPs

The FTIR spectrum of the prepared AuNPs is presented in Fig. 4. The NPs are prepared by citrate reduction that have characteristic bands of citrate at 3429.78, 1244.83, 609.39, and 1078 cm^−1^ as shown in Fig. 4. These bands were assigned to the stretching vibration of the OH group, C-O stretching, C = O stretching, and CO–O-C symmetric stretching, respectively. The main characteristic peaks for AuNPs are shown in Table 2.

**Table 2:** Assignment of the FTIR spectra of gold nanoparticles presented in Fig. 4

AuNPs	
Fundamental vibrations	Frequency (cm^−1^)
O–H stretch	3429.78
Asymmetric C-H stretching	2923.56
C-H stretching	2365.26
C = C stretching	1612.2
C–OH in plane bending	1417.42
C-O stretching	1244.83
CO–O-C symmetric stretching	1078.01
NH_2_ wagging and twisting	837.91
C = O stretching	609.39

### The effect of AuNPs on cell viability of oral epithelial cell line

**Fig. 5:**
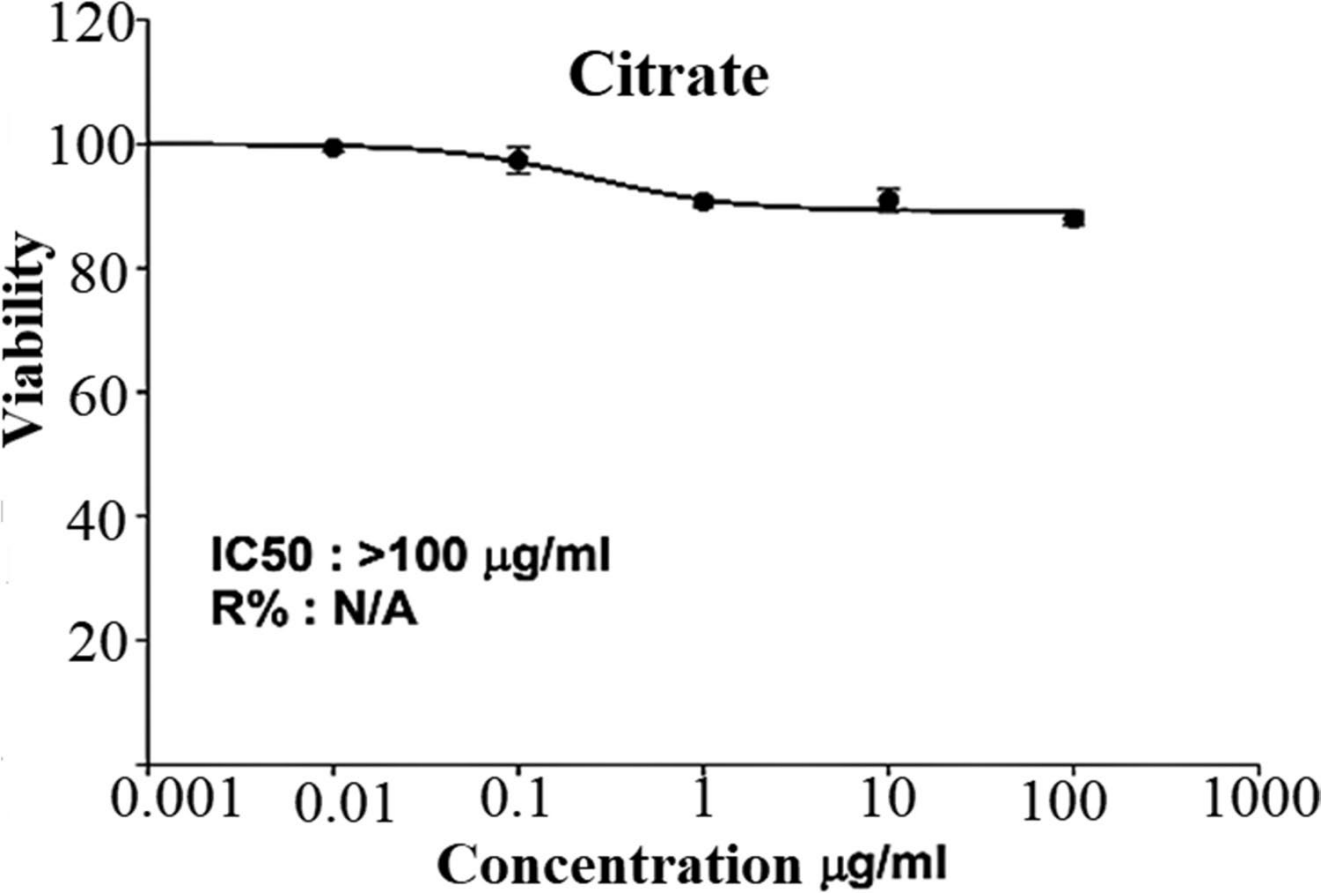
Cytotoxicity test at different concentrations (µg/ml) of AuNPs on oral epithelial cell line after incubation for 72 h

In order to evaluate AuNP potential for biomedical application, cytotoxicity of AuNPs was evaluated against oral epithelial cell line (OEC) at different concentrations after 72 h using the SRP assay. AuNPs induced a concentration-dependent cell viability reduction reaching maximum 13% at 100 μg/ml (see Fig. 5) which reflects the safety of AuNPs on normal oral epithelial cell line. The main cause of this cytotoxicity was the induction of apoptosis [22].

### Laser-induced fluorescence

**Fig. 6:**
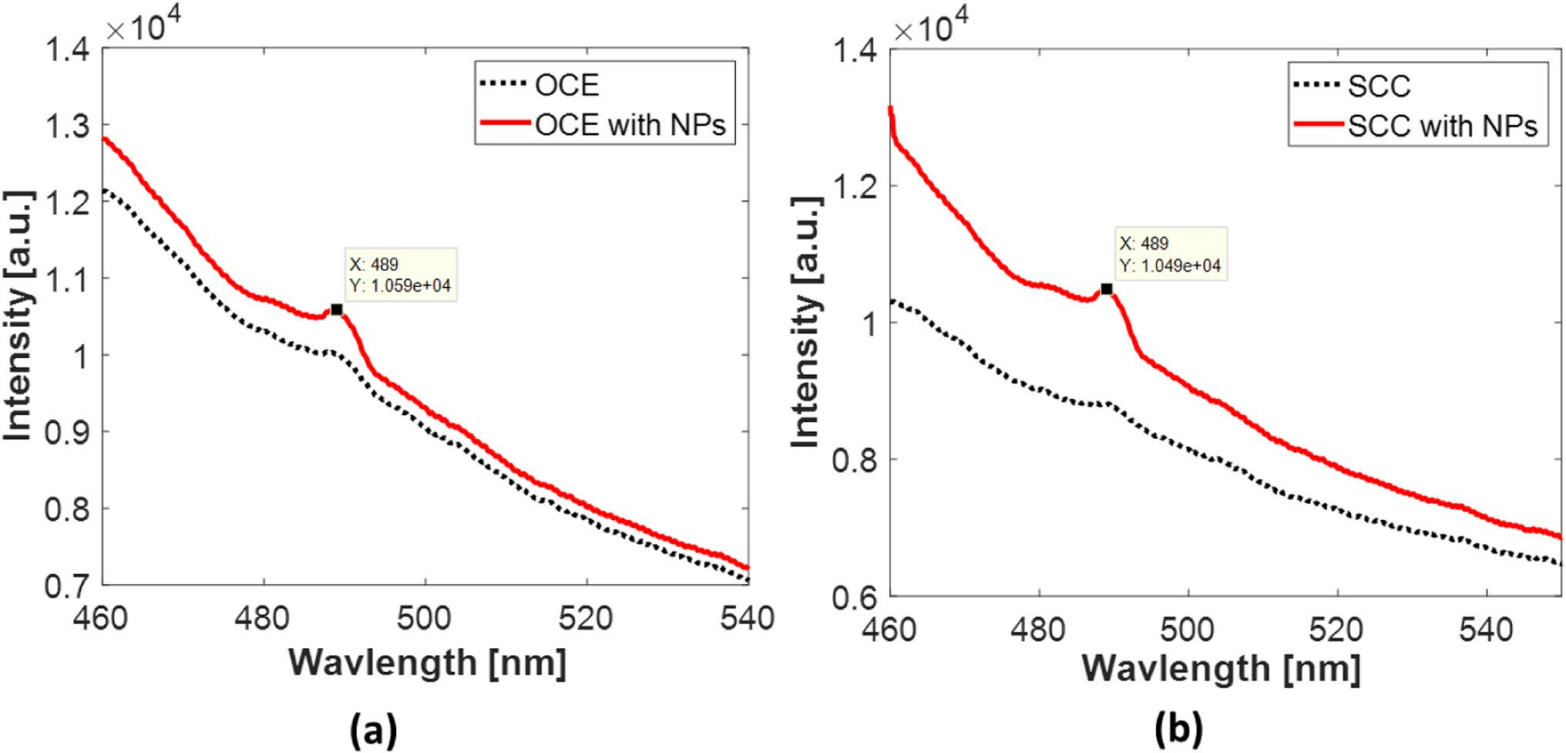
**a**, **b** LIF spectra of normal and cancer cells before and after adding the nanoparticles

Upon exciting the examined samples by the 405-nm incident laser irradiation, a fluorescence peak was observed at 489 nm in the OCE and SCC mixed with AuNPs as presented in Fig. 6. However, this peak is relatively higher in the SCC mixed with AuNPs compared with those without adding the NPs.

### Reflection analysis

**Fig. 7:**
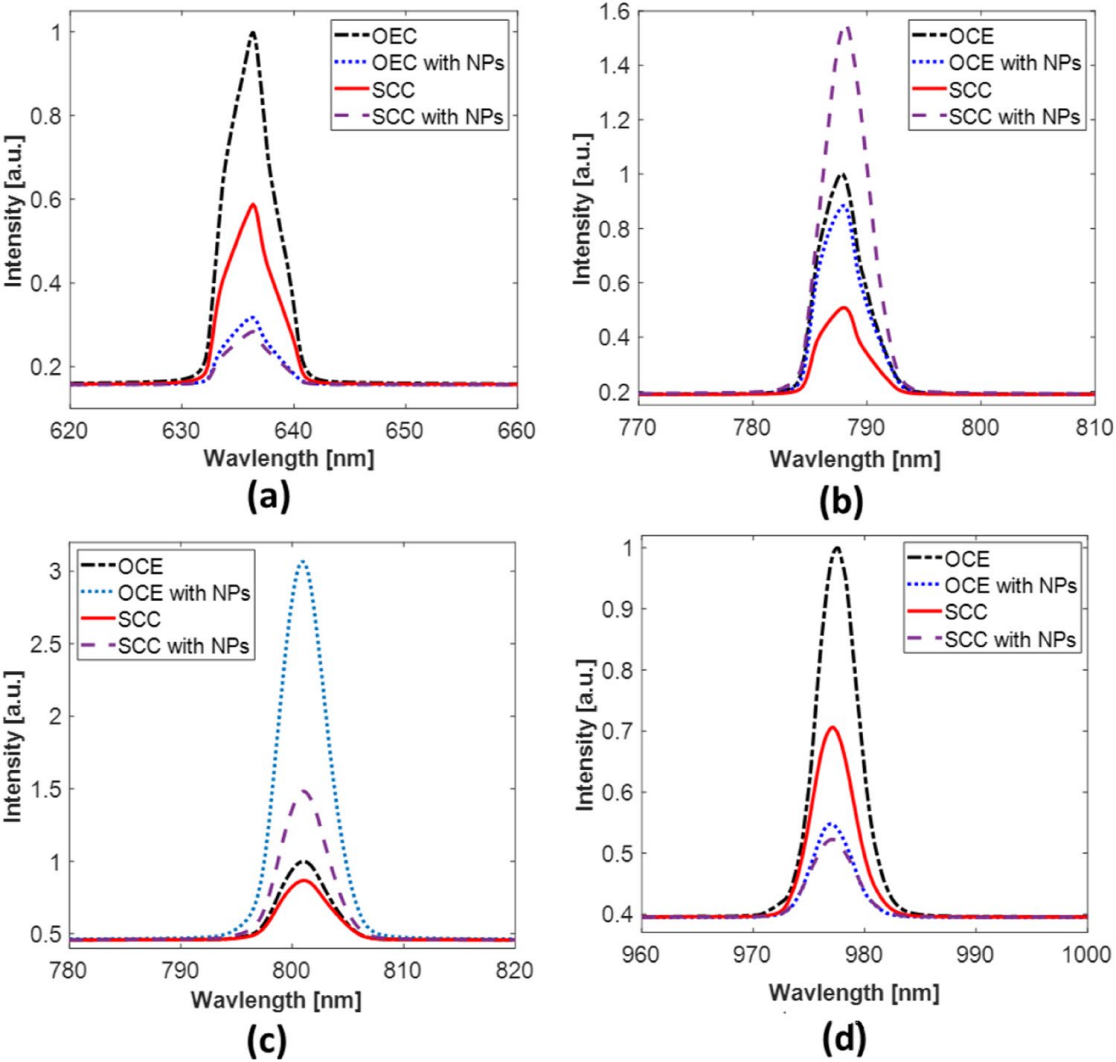
Optical reflectance measurement at different laser wavelengths: (**a**) 635 nm, (**b**) 785 nm, (**c**) 808 nm, (**d**) 980 nm

Figure 7a  shows low reflection in squamous cell carcinoma with AuNPs and also in normal epithelial cells with AuNPs; on the other hand, it shows high reflection in squamous cell carcinoma and normal epithelial cells without AuNPs at 635 nm. Almost similar results were obtained at the 980-nm laser irradiation (Fig. 7d). Moreover, Fig. 7b  shows a high reflection in squamous cell carcinoma with AuNPs and also moderate reflection in normal epithelial cells with and without AuNPs in addition to low reflection in squamous cell carcinoma without AuNPs at 785 nm. At 808 nm, the highest reflectance was obtained in the OCE with NPs, while the SCC showed the lowest one. Additionally, moderate reflectance in SCC with NPs was observed (see Fig. 7c).

**Fig. 8:**
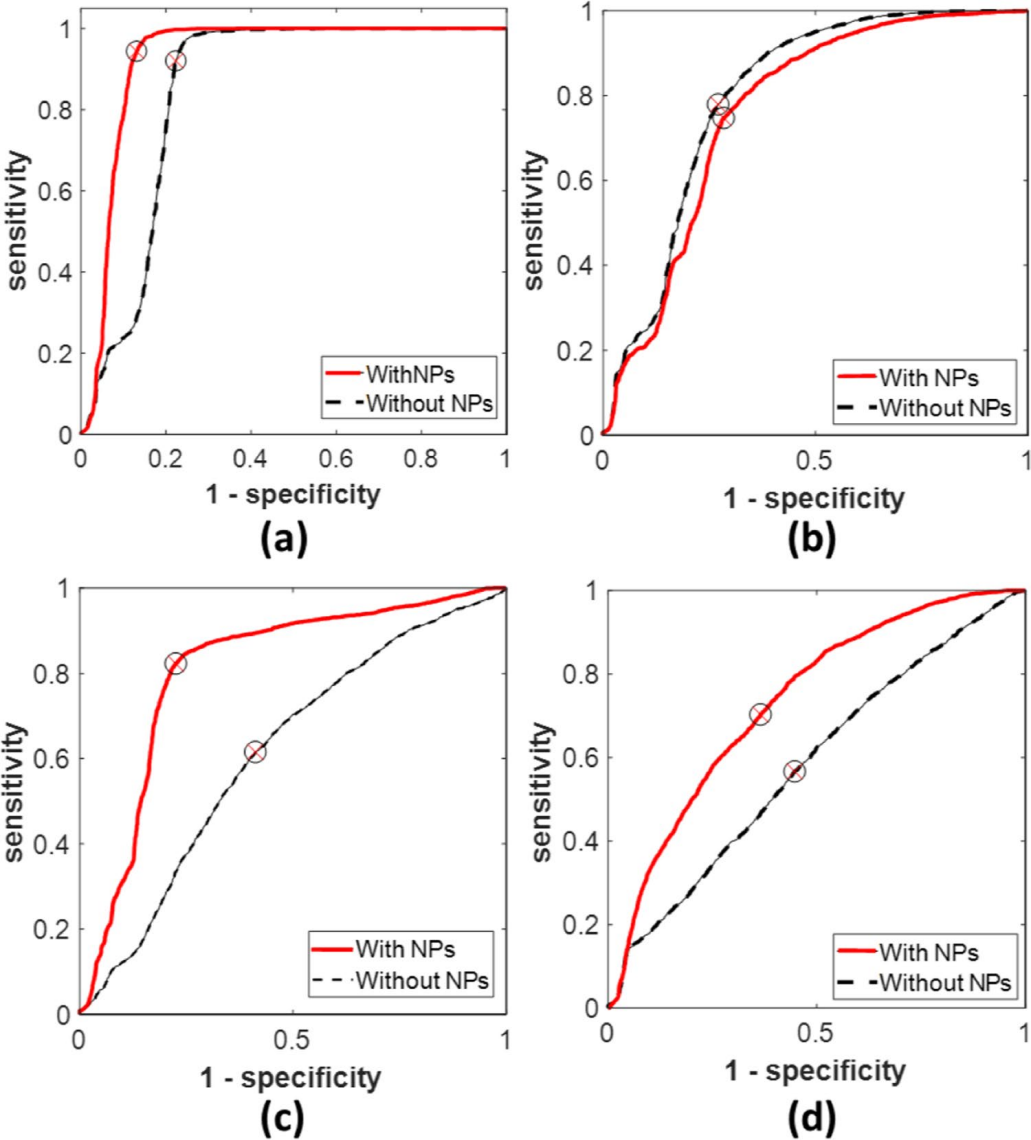
ROC curves of the studied cell lines before and after adding NPs: (**a**) at 660 nm, (**b**) at 785 nm, (**c**) at 808 nm, (**d**) at 980 nm

For evaluating the sensitivity and accuracy of the proposed results, the receiver operating characteristic (ROC) curves at the selected wavelengths were created before and after using the AuNPs as illustrated in Fig. 8. The obtained area under the ROC curve “AUC” is the key parameter for evaluation [23, 24]. As presented in Fig. 8, the highest AUC (93%) was obtained at 635 nm for the SCC mixed with NPs. Moreover, the area under the ROC curves were significantly increased after adding the NPs to the SCC samples using 808-nm and 980-nm lasers as presented in Fig. 8c, d, respectively. On the other hand, no significant enhancement was observed at 785 nm. The whole ROC parameters are summarized in Table 3.

**Table 3:** The ROC curve parameters at every studied wavelength

Wavelength (nm)	Sensitivity		Specificity		AUC		Accuracy	
	Without NPs	With NPs	Without NPs	With NPs	Without NPs	With NPs	Without NPs	With NPs
660	0.92	0.94	0.78	0.87	0.85	0.93	0.85	0.91
785	0.78	0.75	0.73	0.72	0.80	0.77	0.75	0.73
808	0.62	0.82	0.59	0.77	0.61	0.81	0.60	0.80
980	0.57	0.70	0.55	0.65	0.58	0.73	0.56	0.70

## Discussion

Referring to Fig. 3, the negative value of zeta potential was due to the presence of three deprotonated anionic carboxyl groups of citrate ions which verify the repulsive interaction between nanoparticles and aiming to prevent the agglomeration of AuNPs. It is noted that size estimated from TEM microscopy is smaller than that obtained from the dynamic light scattering method. This can be explained in terms of the lack of the hydration shell during the TEM particle size determination [25, 26]. Moreover, in the FTIR spectrum of the utilized NPs (Fig. 4), the recorded peaks indicate the involvement of carboxylate ions of citrate in AuNP reduction [27]. Additionally, the prepared AuNPs were safe on the normal oral epithelial cell line with 13% concentration-dependent cell viability reduction at 100 μg/ml as confirmed in Fig. 5.

Unlike the non-metallic nanoparticles, the AuNPs show a high reflection in intense color due to their photo-physical action when exposed to light. The oscillating electromagnetic field of the light shows a collective coherent oscillation of the free electron of the metal. This electron oscillation around the particle surface causes a charge separation with respect to the ionic lattice, forming a dipole oscillation along the direction of the electric field of the light; the amplitude of the oscillation reaches maximum at a specific frequency called surface plasmon resonance (SPR). The SPR makes a strong absorption of light and thus can measured using a UV and visible light. Moreover, the SPR is very strong for plasmonic nanoparticles especially AuNPs than other metals. AuNPs show its peak at 520 nm [28]. Accordingly, the AuNPs have been widely used as a contrast agent in various cancer diagnostics techniques including MRI, CT, and fluorescence imaging [29].

The fluorescence and diffuse reflection analysis revealed different absorption and scattering properties of the normal and cancerous cell lines before and after adding the NPs. However, some wavelengths showed more significant insights of discrimination than others due to the sample (cells/tissues) optical properties (i.e., absorption and scattering) dependency on the utilized laser wavelength [30, 31]. Compared to the literature, our obtained results at 785 nm are in good agreement with the literature that used a near wavelength (780 nm) in which a high reflection was also observed at the areas identified histologically as SCC after adding AuNPs [32].

## Conclusion

In conclusion, laser-induced fluorescence and diffuse reflectance analysis has been utilized to reveal the improvement in diagnosing the SCC of the tongue after mixing with AuNPs. A fluorescence emission (489 nm) has been obtained using an excitation laser source (405 nm) after adding the nanoparticles. The diffuse reflectance of the studied samples has been obtained and is compared at various laser wavelengths (635, 785, 808, and 980 nm) before and after adding the AuNPs. For results evaluation, the ROC curves at each case have been created and the resultant parameters have been assessed. The obtained results reveal the potential of using AuNPs combined with fluorescence and reflectance measurements for the efficient detection of the SCC of the tongue.
